# Performance Comparison with Different Antenna Properties in Time Reversal Ultra-Wideband Communications for Sensor System Applications

**DOI:** 10.3390/s18010088

**Published:** 2017-12-30

**Authors:** Yu Yang, Bing-Zhong Wang, Shuai Ding

**Affiliations:** Institute of Applied Physics, University of Electronic Science and Technology of China, Chengdu 610054, China; yuyanghd@gmail.com (Y.Y.); uestcding@hotmail.com (S.D.)

**Keywords:** UWB antenna, time reversal, channel measurement, channel capacity

## Abstract

The complexity reduction of receivers in ultrawideband (UWB) communication when time reversal (TR) technique is applied makes it suitable for low-cost and low-power sensor systems. Larger antenna dispersion can generally lead to a less stable phase center and will increase the interference in UWB communications based on pulse radio, whereas a higher antenna gain will result in higher channel gain and further larger channel capacity. To find out the trade-off between antenna gain and dispersion, we performed the channel measurements using different antennas in a dense multipath environment and established the distribution of channel capacities based on the measured channel responses. The results show that the capacity loss caused by antenna dispersion cannot be compensated by antenna gain with line-of-sight transmission to some extent, the effect of phase center on the communication system is negligible, and antennas with smaller time dispersion will have a better energy focusing property and anti-interference performance in TR systems.

## 1. Introduction

Ultra-wideband (UWB) has been applied in various wireless sensor networks (WSNs) due to their low complexity, low cost, low interference and high time domain resolution [1]. One central issue facing the UWB community is how to effectively collect energy, which is dispersed in a rich multipath. Time reversal (TR) is a self-adaptive technique that can be used to compensate for any geometrical and channel distortions due to the propagation through inhomogeneous media [2]. Moreover, it has been proved to be an ideal paradigm in suppressing the time delay spread caused by the rich multipath, and an extremely simple non-coherent receiver can be used for low-cost and low-power sensors based on the TR technique [3,4].

In UWB radio wave propagations, the multiple scattering effect contributes to the temporal dispersion of the pulsed shaped transmit signals and the multiple diffraction of incident wave leads to the distortion of the incident wave [5]. An accurate uniform theory of diffraction (UTD) model for the analysis of complex indoor radio environments is presented by taking into account the effects of building floors, walls, windows, and the presence of metallic and penetrable furniture into account [6]. Moreover, the measurement and modelling of spatial channel, such as indoor channels, warehouse environments, parked cars in underground garages, and foliage environments have also investigated the propagation of UWB signals [7,8,9,10]. The efficient algorithm for channel response extraction such as cluster identification and the power delay profile (PDP) model have also been developed [11].

In a practical pulse radio UWB system, the parameters and positions of antennas will significantly influence the power gain of the transmission link, thus the radiation properties of mounted antennas in UWB communication system must be considered in the system deployment [12,13]. The pulse distortion of the UWB antennas will inevitably reduce the system performance, and physical or numerical methods can compensate this kind of distortion [14,15], but they are complex and not appropriate for some low-cost and low-power sensors system. Furthermore, waves propagating through dense multipath environment such as the inner space of a closed cavity (vehicle, airplane, spacecraft etc.) will lead to time-spreading of pulses and make signal transmission less predictable and less reliable in sensors system [16,17]. The TR technique can adaptively compensate pulse distortion with a simultaneous spatial-temporal focusing property; thus, it is necessary to investigate the antenna and radio propagation performance in a UWB sensors system based on a TR technique when deployed in complex environments.

It is well known that the time dispersion property of a UWB antenna will decrease the signal-to-noise ratio (SNR) of the UWB communication system, and antennas with larger time dispersion generally have less stable phase centers [18]. The directive antenna generally has larger antenna gain than the omnidirective antenna and will produce a larger channel gain in line-of-sight transmission, while some directive antennas also have worse dispersion properties, which means larger interference in communication than the omnidirective antenna [19]. Although some antennas such as the transverse electromagnetic wave (TEM) horn antenna, ridged horn and dielectric rod antenna share high gain and low dispersion properties simultaneously, they are not easily integrated in UWB sensors’ application. Therefore, how to achieve a balance between the antenna dispersion and antenna gain in a specific deployment using easily integrated planar antennas deserves attention.

In order to explore the trade-off between antenna gain and time dispersion property in TR based UWB communication systems, we use three kinds of typical planar antennas (i.e., monopole, log-periodic and Vivaldi antenna) with different radiation patterns and transfer properties to measure the channel response and then to evaluate the system performance. The measurements are deployed in a closed metal cavity that is used to simulate an enclosing cabin and produce multipaths. Results show that the larger antenna gain cannot effectively compensate for the capacity loss caused by the antenna dispersion to a certain extent in TR UWB communication, and the impact of phase center on the channel capacity is not obvious. In addition, antennas with smaller time dispersion have better energy focusing and anti-interference performance in TR UWB systems.

The rest of this paper is organized as follows. In Section 2, the theoretical foundation of UWB communication based on TR in WSNs is presented and the measured parameters are defined. The characteristic parameters of antennas are displayed in Section 3. Section 4 introduces the experimental setup in detail. The measured results and discussions are addressed in Section 5. Section 6 concludes the whole paper.

## 2. Theoretical Foundation of Time Reversal UWB Communication

**Figure 1:**
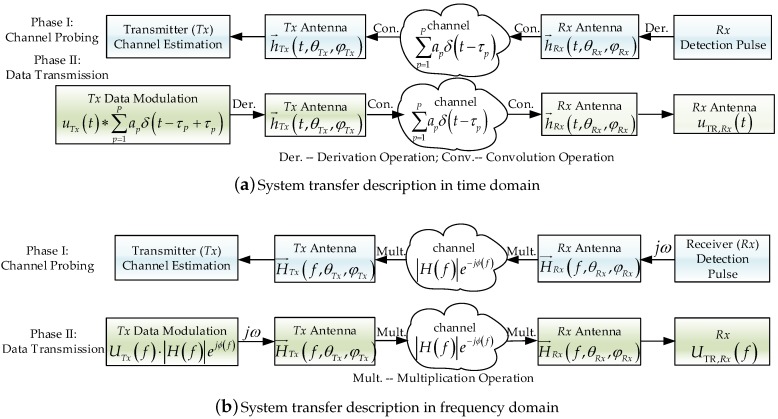
Systemtransfer schemes of time reversal based ultra-wideband (TRUWB) communication system, (**a**) in time domain; (**b**) in frequency domain.

The basic principle of pulse-based TR UWB communication in WSNs is as follows. In the first phase, the receiver (*Rx*) antenna sends a probing signal to the transmitter (*Tx*) antenna and the *Tx* will estimate the channel response. In the second phase, the *Tx* antenna transmits information symbols that are modulated by the time-reversed channel response [3]. The complete transmission link including antenna effects and spatial propagation in frequency domain based on channel reciprocity is derived and the equivalent TR channel response is also obtained. The complete transfer structure of the TR UWB system in time domain is presented in Figure 1a and the transfer scheme in frequency domain is presented in Figure 1b . When the signal is transmitted through a UWB antenna, the antenna output signal contains the input signal and its derivatives with varying delays, caused mainly by the resonances in the radiator structure. The radiated electromagnetic fields are derived from the differentiation of the current/charge distribution on the antenna [18].

The parameters used in the time-domain link description are: amplitude of *Tx* signal $u_{Tx}\left(t\right)$ in [V]; amplitude of *Rx* signal $u_{Rx}\left(t\right)$ in [V]; angle-of-departure of ray at the *Tx* antenna $\left(\theta_{Tx},\varphi_{Tx}\right)$; angle-of-arrival of ray at the *Rx* antenna $\left(\theta_{Rx},\varphi_{Rx}\right)$; amplitude of the equivalent TR receive signal $u_{\mathrm{TR},Rx}\left(t\right)$ in [V]; transfer function of *Tx* antenna ${\vec{h}}_{Tx}\left(t,\theta_{Tx},\varphi_{Tx}\right)$; transfer function of *Rx* antenna ${\vec{h}}_{Rx}\left(t,\theta_{Rx},\varphi_{Rx}\right)$; characteristic impedance of *Tx* antenna $Z_{c,Tx}$ in [Ω]; characteristic impedance of *Rx* antenna $Z_{c,Rx}$ in [Ω]; wave impedance of the free space $Z_{0}$ in [Ω]. In addition, the corresponding descriptions in the frequency-domain link description are: amplitude of *Tx* signal $U_{Tx}\left(f\right)$ in [V]; amplitude of *Rx* signal $U_{Rx}\left(f\right)$ in [V]; amplitude of the received equivalent TR signal $U_{\mathrm{TR},Rx}\left(f\right)$ in [V]; transfer function of *Tx* antenna ${\vec{H}}_{Tx}\left(f,\theta_{Tx},\varphi_{Tx}\right)$; transfer function of *Rx* antenna ${\vec{H}}_{Rx}\left(f,\theta_{Rx},\varphi_{Rx}\right)$.

Assuming the total number of the transmission paths is *P*, then the incident signal of the *p*th path with amplitude fading factor $a_{p}$ and path delay $\tau_{p}$ at the receiver is modeled as follows:

(1) $$\frac{{\vec{e}}_{Tx,p}\left(t,\theta_{Tx,p},\varphi_{Tx,p}\right)}{\sqrt{Z_{0}}}=a_{p}\delta \left(t-\tau_{p}\right)*{\vec{h}}_{Tx}\left(t,\theta_{Tx,p},\varphi_{Tx,p}\right)*\frac{\partial }{\partial t}\frac{u_{Tx}\left(t\right)}{\sqrt{Z_{c,Tx}}}$$

where δ(t) is the Dirac delta function and * denotes the convolution operator. The transfer function of the *Tx* antenna is ${\vec{h}}_{Tx}\left(t,\theta_{Tx},\varphi_{Tx}\right)=h_{Tx,copol}\left(t,\theta_{Tx},\varphi_{Tx}\right){\vec{e}}_{copol}+h_{Tx,xpol}\left(t,\theta_{Tx},\varphi_{Tx}\right){\vec{e}}_{xpol}$, it contains the co-polar component $h_{Tx,copol}$ and cross-polar component $h_{Tx,xpol}$ of the antenna transfer function, ${\vec{e}}_{copol}$ is the unit vector in the co-polar direction and ${\vec{e}}_{xpol}$ is the unit vector in the cross-polar direction. Since the channel is reciprocal, the linearity of the problem allows for the superposition of all incident plane waves at the *Rx* antenna, and then the total received electrical signal at the *Rx* antenna can can be expressed as

(2) $$\frac{{\vec{e}}_{Tx}\left(t\right)}{\sqrt{Z_{0}}}=\sum_{p=1}^{P}a_{p}\delta \left(t-\tau_{p}\right)*{\vec{h}}_{Tx}\left(t,\theta_{Tx,p},\varphi_{Tx,p}\right)*\frac{\partial }{\partial t}\frac{u_{Tx}\left(t\right)}{\sqrt{Z_{c,Tx}}}$$

Then, the received signal at the *Rx* end is the convolution of the incident signal with the *Rx* transfer function, which is denoted as

(3) $$\frac{u_{Rx}\left(t\right)}{\sqrt{Z_{c,Rx}}}={\vec{h}}_{Rx}\left(t,\theta_{Rx,p},\varphi_{Rx,p}\right)*\frac{{\vec{e}}_{Tx}\left(f,\theta ,\varphi \right)}{\sqrt{Z_{0}}}$$

where ${\vec{h}}_{Rx}\left(t,\theta_{Rx},\varphi_{Rx}\right)=h_{Rx,copol}\left(t,\theta_{Rx},\varphi_{Rx}\right){\vec{e}}_{copol}+h_{Rx,xpol}\left(t,\theta_{Rx},\varphi_{Rx}\right){\vec{e}}_{xpol}$ is the transfer function of the *Rx* antenna. Substitute (2) into (3) can obtain the expression of the *Rx* signal as:

(4) $$\frac{u_{Rx}\left(t\right)}{\sqrt{Z_{c,Rx}}}=\sum_{p=1}^{P}a_{p}\delta \left(t-\tau_{p}\right)*{\vec{h}}_{Rx}\left(t,\theta_{Rx,p},\varphi_{Rx,p}\right)*{\vec{h}}_{Tx}\left(t,\theta_{Tx,p},\varphi_{Tx,p}\right)*\frac{\partial }{\partial t}\frac{u_{Tx}\left(t\right)}{\sqrt{Z_{c,Tx}}}$$

The system transfer function in time domain is obtained in (4), after Fourier Transform, the equivalent system transfer function in frequency domain can also be expressed as

(5) $$\frac{U_{Rx}\left(f\right)}{\sqrt{Z_{c,Rx}}}=\sum_{p=1}^{P}A_{p}e^{-j\phi_{p}}{\vec{H}}_{Rx}\left(f,\theta_{Rx,p},\varphi_{Rx,p}\right)\cdot {\vec{H}}_{Tx}\left(f,\theta_{Tx,p},\varphi_{Tx,p}\right)j\omega \frac{U_{Tx}\left(f\right)}{\sqrt{Z_{c,Tx}}}$$

where $A_{p}$ is the amplitude fading factor and $\phi_{p}=2\pi f\tau_{p}$ is the phase shift. The total channel response in frequency domain with the antenna transfer functions and the fading effects in the free space included is thus obtained in (5).

In TR UWB communication, channel estimation is in the first phase, and then the time-reversed version of the estimated channel response will be used to modulate the transmitted signal $u_{sig}\left(t\right)$ in the second phase. Since the channel is reciprocal, the input signal of the *Tx* antenna in TR UWB communication can be written as

(6) $$u_{\mathrm{TR},Tx}\left(t\right)=\sum_{p=1}^{P}a_{p}\delta \left(t+\tau_{p}-\tau_{P}\right)*{\vec{h}}_{Rx}\left(\tau_{P}-t,\theta_{Rx,p},\varphi_{Rx,p}\right)*{\vec{h}}_{Tx}\left(\tau_{P}-t,\theta_{Tx,p},\varphi_{Tx,p}\right)*u_{sig}\left(t\right)$$

where $\tau_{P}$ is the maximal path delay of the channel response, due to the path delay being generally larger than the time dispersion of the antenna transfer function. The antenna transfer functions ${\vec{h}}_{Tx}\left(\tau_{P}-t,\theta ,\varphi \right)$ and ${\vec{h}}_{Rx}\left(\tau_{P}-t,\theta ,\varphi \right)$ are the time reversed version of the antenna transfer function with stationary time delay. Similar to the process in (2), the total incident signal at the *Rx* antenna is

(7) $$\frac{{\vec{e}}_{\mathrm{TR},Tx}\left(t\right)}{\sqrt{Z_{0}}}=\sum_{p=1}^{P}a_{p}\delta \left(t-\tau_{p}\right)*{\vec{h}}_{Tx}\left(t,\theta_{Tx,p},\varphi_{Tx,p}\right)*\frac{\partial }{\partial t}\frac{u_{\mathrm{TR},Tx}\left(t\right)}{\sqrt{Z_{c,Tx}}}$$

Similar to the derivation of (4), the received signal at the *Rx* end in the TR communication is

(8) $$\frac{u_{\mathrm{TR},Rx}\left(t\right)}{\sqrt{Z_{c,Rx}}}=\sum_{p=1}^{P}a_{p}\delta \left(t-\tau_{p}\right)*{\vec{h}}_{Rx}\left(t,\theta_{Rx,p},\varphi_{Rx,p}\right)*{\vec{h}}_{Tx}\left(t,\theta_{Tx,p},\varphi_{Tx,p}\right)*\frac{\partial }{\partial t}\frac{u_{\mathrm{TR},Tx}\left(t\right)}{\sqrt{Z_{c,Tx}}}$$

Substituting (6) into (8), and assuming the characteristic impedances $Z_{c,Tx}$ and $Z_{c,Rx}$ are equal, the equivalent TR channel response in the time domain can be written as

(9) $$\begin{array}{c} h_{\mathrm{TR}}\left(t\right)=\sum_{p=1}^{P}{\left|a_{p}\right|}^{2}\delta \left(t-\tau_{P}/2\right)*corr\left\{{\vec{h}}_{Tx}\left(t,\theta_{Tx},\varphi_{Tx}\right)\right\}*corr\left\{{\vec{h}}_{Rx}\left(t,\theta_{Rx},\varphi_{Rx}\right)\right\}\\ +\sum_{p=1}^{P}\sum_{l=1,l\neq p}^{P}\delta \left(t-\tau_{p}\right)\delta \left(t+\tau_{l}-\tau_{P}\right)*xorr\left\{{\vec{h}}_{Tx}\left(t,\theta_{Tx},\varphi_{Tx}\right)\right\}*xorr\left\{{\vec{h}}_{Rx}\left(t,\theta_{Rx},\varphi_{Rx}\right)\right\} \end{array}$$

In (9), corr{·} is the auto-correlation function, i.e., $corr\left\{{\vec{h}}_{Tx}\left(t,\theta_{Tx},\varphi_{Tx}\right)\right\}={\vec{h}}_{Tx}\left(t,\theta_{Tx,p},\varphi_{Tx,p}\right)*{\vec{h}}_{Tx}\left(\tau_{P}-t,\theta_{Tx,p},\varphi_{Tx,p}\right)$. Similarly, $xorr\left\{{\vec{h}}_{Tx}\left(t,\theta_{Tx},\varphi_{Tx}\right)\right\}={\vec{h}}_{Tx}\left(t,\theta_{Tx,p},\varphi_{Tx,p}\right)*{\vec{h}}_{Tx}\left(\tau_{P}-t,\theta_{Tx,l},\varphi_{Tx,l}\right)$ is the mutual correlation function. |·| represents the absolute value of the variable, ∥·∥ is the module of the vector, and ${\left(\cdot \right)}^{*}$ is the conjugate of the variable. The equivalent TR channel response $H_{\mathrm{TR}}\left(f\right)$ in (9) can be turned into frequency domain by Fourier Transform, and it contains two parts: the signal component $S_{gain}\left(f\right)$ and the interference component $S_{int}\left(f\right)$. Their expressions are:

(10) $$\begin{array}{cc} & H_{\mathrm{TR}}\left(f\right)=S_{gain}\left(f\right)+S_{int}\left(f\right) \end{array}$$

(11) $$\begin{array}{cc} & S_{gain}\left(f\right)=\sum_{p=1}^{P}{\left|A_{p}\right|}^{2}{∥{\vec{H}}_{Rx}\left(f,\theta_{Rx,p},\varphi_{Rx,p}\right)∥}^{2}{∥{\vec{H}}_{Tx}\left(f,\theta_{Tx,p},\varphi_{Tx,p}\right)∥}^{2} \end{array}$$

(12) $$\begin{array}{c} S_{int}\left(f\right)=\sum_{p=1}^{P}\sum_{l=1,l\neq p}^{P}A_{p}A_{l}{\vec{H}}_{Rx}\left(f,\theta_{Rx,p},\varphi_{Rx,p}\right)\cdot {\vec{H}}_{Tx}\left(f,\theta_{Tx,p},\varphi_{Tx,p}\right)\\ {\vec{H}}_{Rx}^{*}\left(f,\theta_{Rx,l},\varphi_{Rx,l}\right)\cdot {\vec{H}}_{Tx}^{*}\left(f,\theta_{Tx,l},\varphi_{Tx,l}\right) \end{array}$$

In (10)–(12), it can be seen that the equivalent TR channel response depends on the spatial multipath transmission as well as the antenna transfer functions both at the *Tx* and *Rx* end. The spatial channel response and antenna transfer functions can all be measured precisely in the time domain or frequency domain.

## 3. Characteristic Parameters of Antennas

**Figure 2:**
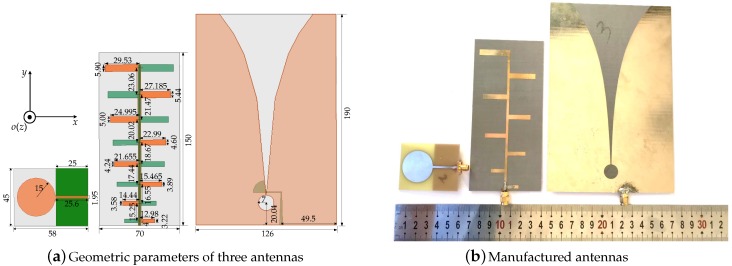
Specific structures of three antennas, from left to right are monopole, log-periodic and Vivaldi antennas, respectively.

The three typical kinds of planar antennas that are used to measure the channel responses of TR UWB sensors system are displayed in Figure 2. Figure 2a shows the geometric parameters of each antenna, the numerical unit is millimeters (mm), and from left to right are monopole, log-periodic and Vivaldi antenna, respectively. The selected reference axis system is also presented, the angle θ starts from the positive *z*-axis and the angle φ starts from the positive *x*-axis as usual. The orange structure on the front side of the monopole is the metal radiation patch and the olive structure on the backside is the metal ground. The metal orange structure on the front side and the metal olive structure on the backside of the log-periodic antenna are totally symmetrical. The light orange color on the front side of the Vivaldi antenna is the radiating structure with tapered slot and the dark yellow structure on the backside is the feed balun. The slot curve of the Vivaldi antenna is exponential function, which is expressed as $x=e^{\left(0.02*y\right)}-0.16$. The light gray parts are substrate plates and the thickness of three substrate plates are all 1 mm. The permittivities of the monopole, log-periodic and Vivaldi antenna are 4.4, 2.65 and 3.5, respectively. The manufactured antennas are shown in Figure 2b, the sizes of the monopole, log-periodic and the Vivaldi antenna are 58 mm ×45 mm, 70 mm ×150 mm and 126 mm ×190 mm, respectively. It can be seen that the effective area of the Vivaldi antenna is the largest.

### 3.1. Radiation Properties of Antennas

**Figure 3:**
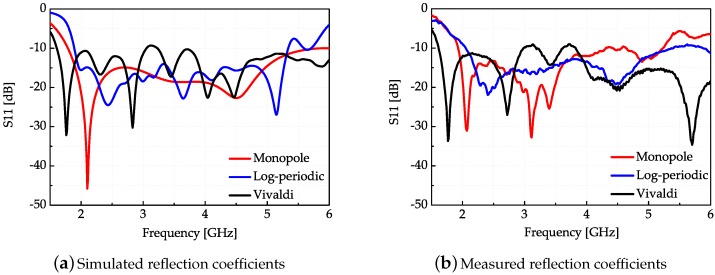
Simulated and measured reflection coefficients of three antennas.

**Figure 4:**
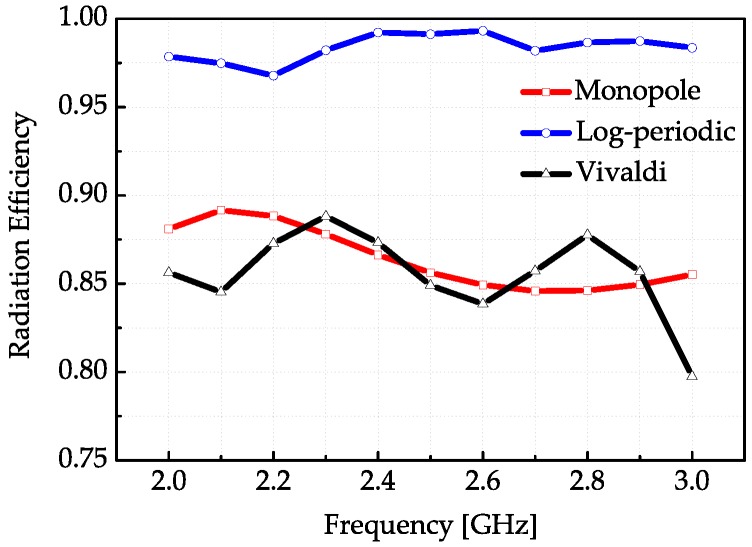
Radiation efficiencies of the three antennas in the measured bandwidth.

**Figure 5:**
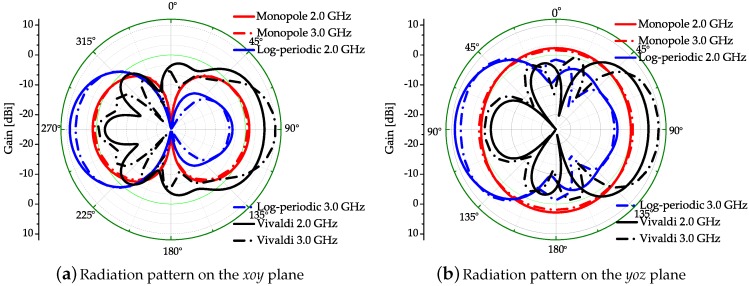
Radiation patterns of three antenna on different planes.

The simulated and measured S-parameters of each antenna are correspondingly shown in Figure 3. It can be seen that the simulated and measured curves are matched well under 6 GHz. In this experiment, the frequency bandwidth selected is the common working bandwidth of three antennas from 2 GHz to 3 GHz. Figure 4 displays the radiation efficiency of each antenna. The radiation efficiency of the log-periodic antenna in the measurement bandwidth is the highest. Figure 5a presents the radiation patterns of the three antennas on the *xoy* plane and Figure 5b are the radiation patterns on the *yoz* plane (the axis system has been shown in Figure 2a), respectively. The radiation pattern of the monopole on the *yoz* plane is nearly omnidirectional, while the log-periodic and Vivaldi antenna are directive. The maximum gain and 3 dB beamwidth of these three antennas at frequencies 2 GHz and 3 GHz have been displayed in Table 1. It can be seen that the gain of the monopole is minimum and the gains of log-periodic and Vivaldi antenna are nearly equal. The 3 dB beamwidth of the log-periodic antenna is larger than the Vivaldi antenna on the *yoz* plane.

### 3.2. Signal Transfer Properties of Antennas

**Figure 6:**
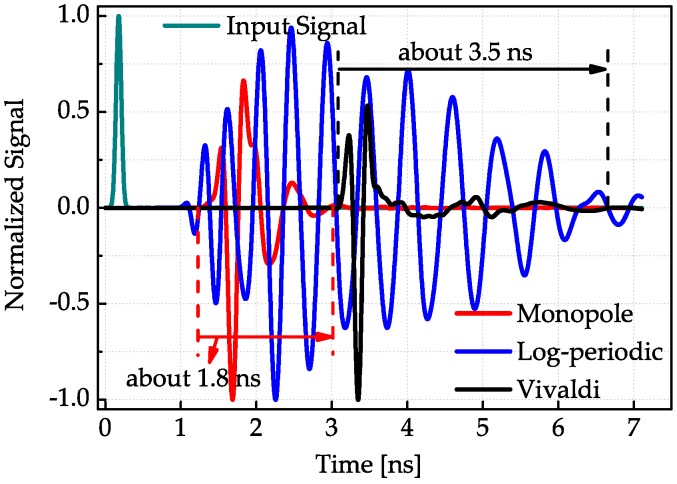
Received signals between the same two antennas in the main lobe direction when the input signals are all first-order Gaussian pulses.

One important parameter that measures the performance of UWB antenna is time dispersion property. The antenna dispersion will result in pulse spread and increase the interference in radio pulse based UWB communications. In order to evaluate the dispersion properties of these three antennas, three simulations are completed in the commercial simulation software CST Microwave Studio 2012 (Computer Simulation Technology, Darmstadt, Germany). The simulation is composed of a pair of the same antennas every time. The main lobe of one antenna is displaced at the main lobe direction of the other antenna with an edge distance being two times the maximum working wavelength. For the Vivaldi antenna, due to its largest size, the absolute distance between two Vivaldi antennas is also the largest, thus the received pulse has the largest transmission time delay. The input signal is a first-order Gaussian pulse while the observed output signals of the other antenna are shown in Figure 6. It can been seen that, in the main lobe direction, the time dispersion of the log-periodic antenna is the largest while the monopole has the smallest pulse spread.

The simulated time dispersion properties are in accordance with the radiation structure of antennas [18]. In order to observe the transfer functions of three antennas in different directions, multiple measurements are deployed using a vector network analyzer (VNA). According to (2)–(4), if the channel has only one path and the distance between two antenna is *r*, then the transfer process in the frequency domain can be expressed as

(13) $$\frac{U_{Rx}\left(f,r\right)}{\sqrt{Z_{c,Rx}}}=\frac{1}{2\pi cr}exp\left(-j\omega \frac{r}{c}\right){\vec{H}}_{Rx}\left(f,\theta_{Rx},\varphi_{Rx}\right)\cdot {\vec{H}}_{Tx}\left(f,\theta_{Tx},\varphi_{Tx}\right)j\omega \frac{U_{Tx}\left(f\right)}{\sqrt{Z_{c,Tx}}}$$

where *c* is the light speed of the free space. The designed characteristic impedances of the same *Tx* and *Rx* antennas are equal; thus, the measured transfer function $S_{21}\left(f\right)$ can be expressed as

(14) $$S_{21}\left(f,r,\theta ,\varphi \right)=\frac{1}{2\pi cr}exp\left(-j\omega \frac{r}{c}\right)j\omega {\vec{H}}_{Rx}\left(f,\theta_{Rx},\varphi_{Rx}\right)\cdot {\vec{H}}_{Tx}\left(f,\theta_{Tx},\varphi_{Tx}\right)$$

Due to the different antenna sizes, the edge distance of 30 cm is chosen for each pair of antennas when the transfer function is measured. The measured results show that the distance only has an impact on the strength of the received signal, and has no impact on the envelope of the received signal. The amplitude of the measured transfer function $S_{21}\left(f\right)$ is first processed by the least-mean-squre (LMS) algorithm to filter out the noise, and then being calculated according to (14). The three antennas are all linear-polarized antennas, thus only the main polarization direction is considered. The *Tx* and *Rx* antennas are reciprocal, which means that the transfer functions of the *Tx* and *Rx* antennas are the same except that the derivation effect of the *Tx* antenna is being additionally considered.

**Figure 7:**
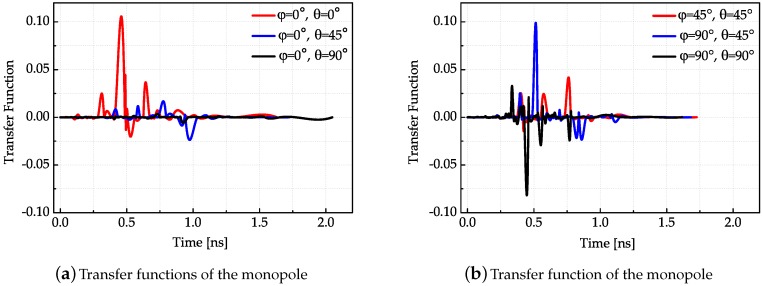
Transfer functions of the monopole in different directions.

**Figure 8:**
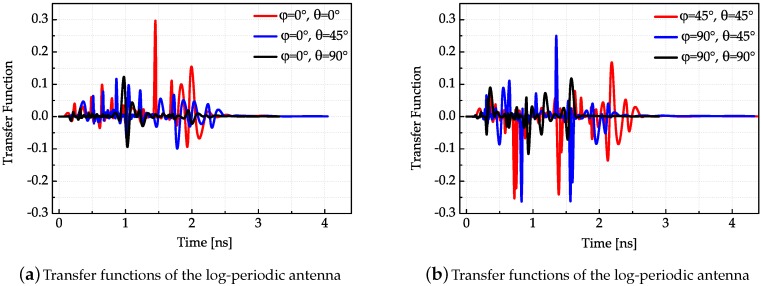
Transfer functions of the log-periodic antenna in different directions.

**Figure 9:**
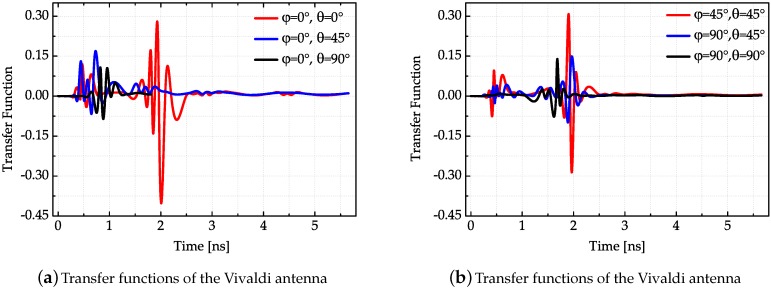
Transfer functions of the Vivaldi antenna in different directions.

After the antenna transfer function in the frequency domain is obtained, the antenna transfer function in the time domain can be acquired by Fourier Inverse Transform. In order to smooth the antenna transfer function, cubic spline interpolation is used to reshape the transfer function. The measured transfer functions in different directions of three antennas are presented in Figure 7, Figure 8 and Figure 9, respectively.

The results shown in Figure 7, Figure 8 and Figure 9 conform to the result in Figure 6. The simulated antenna dispersion properties presented in Figure 6 are in the main lobe direction, and the results in Figure 7, Figure 8 and Figure 9 also demonstrate the difference of antenna transfer functions in different directions, such as signal intensity and antenna dispersion. The amplitudes of the transfer functions corresponding to the same antenna in different directions indicated the directive transmission properties.

**Figure 10:**
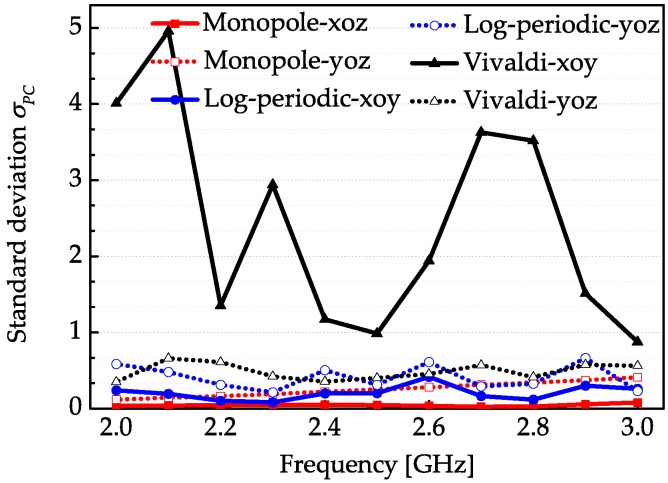
Maximum standard deviation $\sigma_{PC}$ of the phase center locations corresponding to three antennas.

The stability of phase center is also an important parameter for a UWB antenna and the commercial software CST Microwave Studio is used to calculate the phase center of each antenna. The phase centers corresponding to the three antennas are also calculated in the 3 dB angle width of around the maximum radiation direction on corresponding planes [20]. The maximum radiation direction at 2 GHz of the monopole is along the negative direction of the *z*-axis, while the maximum radiation direction of the log-periodic antenna and the Vivaldi antenna are along the negative *y*-axis and positive *y*-axis, respectively. The main lobe direction of the Vivaldi antenna may rotate in a small scale with the frequency variation. We only select two planes to calculate the phase center. The phase centers of the monopole are calculated on the *xoz* plane and *yoz* plane. The phase centers of the log-periodic antenna and the Vivaldi antenna are calculated on the *xoy* plane and *yoz* plane, respectively. Figure 10 presents the maximum standard deviation $\sigma_{PC}$ of the simulated phase center locations corresponding to these three UWB antennas in the working bandwidth. It is obvious that the stabilities of phase center in terms of each antenna on different radiation planes are different. For instance, the stability on the xoy plane of the Vivaldi antenna is much worse than the stability on the *yoz* plane. Moreover, the phase center stability of the monopole is generally the best while the Vivaldi antenna relatively has the worst phase center stability due to its unsymmetrical feed structure and position. Although the log-periodic antenna has larger time spread effect than the Vivaldi antenna, the Vivaldi antenna has a less stable phase center than the log-periodic antenna. It can be concluded that the stability of the phase center in a UWB antenna is related to the radiation mechanisms and the feed structure employed to excite the antenna, and it has no inevitable relationship with the antenna dispersion property.

## 4. Experimental Setup

**Figure 11:**
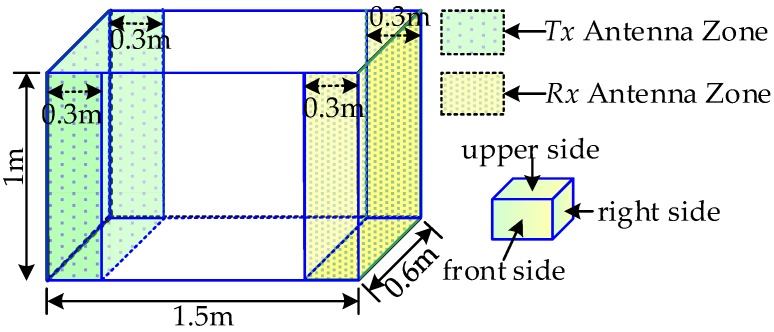
Experimental scene in a closed metal cavity.

All of the measurements are performed in a metal cavity shown in Figure 11. In Figure 11, the left shaded zone of the cavity is where the *Tx* antenna is located and the shaded zone at the right is the location of the *Rx* antenna. The dimension of the cavity is 1.5 m × 0.6 m × 1 m. The *Tx* and *Rx* antennas are independently and randomly placed on the respective location areas. The backward side i.e., the ground of these antennas is attached to the center of a 30 cm × 30 cm foam base with a thickness of 6 cm, and then the foam base is adhered to the metal wall. Firstly, each *Tx* or *Rx* area is divided into uniform grids that have the same areas as the foam with serial numbers. In addition, then the *Tx* or *Rx* is independently placed on the numbered grid according to the pre-generated random number with equal probability. The orientation of the *Tx* and *Rx* antennas are also independently random. No matter which side the antenna is placed on, the positive direction of the *y*-axis (in Figure 2) has four choices and each with probability 1/4. When the antenna is on the front or backward side, the direction of the positive *y*-axis can either be along the upper, lower, left or right side. In the same way, once the antenna is placed on the left or right side, the positive *y*-axis can be along the upper, lower, front or back side. Lastly, the direction of the positive *y*-axis can be along the front, back, left or right side when the antenna is placed on the underside. The measured times of each position will be five and the measured position of each pair of antennas is one hundred.

**Figure 12:**
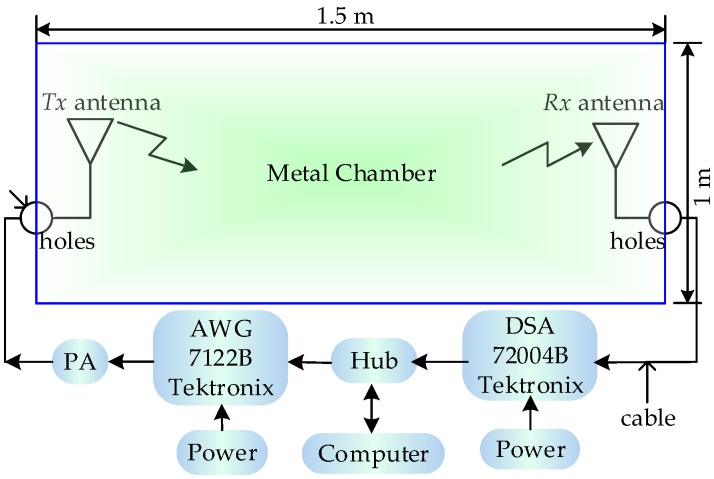
Experimental setup.

This chamber used here can be described as a metal cavity that supports a large amount of resonant cavity modes [21]. Substituting the cavity dimension in this experiment into the calculation of resonant frequency in the resonant cavity, the calculated result shows that there are 2925 modes between the bandwidth 2–3 GHz with a mean frequency interval of 0.34 MHz under the ideal condition. It indicates that almost all waves in measured bandwidth can exist in this cavity. As shown in Figure 12, the experimental process is as follows: firstly, the Arbitrary Waveform Generator (AWG) is used to generate a Gaussian signal with sampling frequency 12 GHz, truncated threshold 10 dB, bandwidth 2–3 GHz and total power of one Watt; subsequently, the signal is amplified by a power amplifier (PA), transmitted through one antenna, propagating in the metal cavity, received by another of the same antenna, and finally recorded by the Digital Serial Analyzer (DSA) with sampling frequency 6.25 GHz. All measurement processes are controlled and operated by the central computer. The *Tx* and *Rx* antennas are connected with the devices using coaxial cables through the holes on the cavity wall.

## 5. Results and Discussion

**Figure 13:**
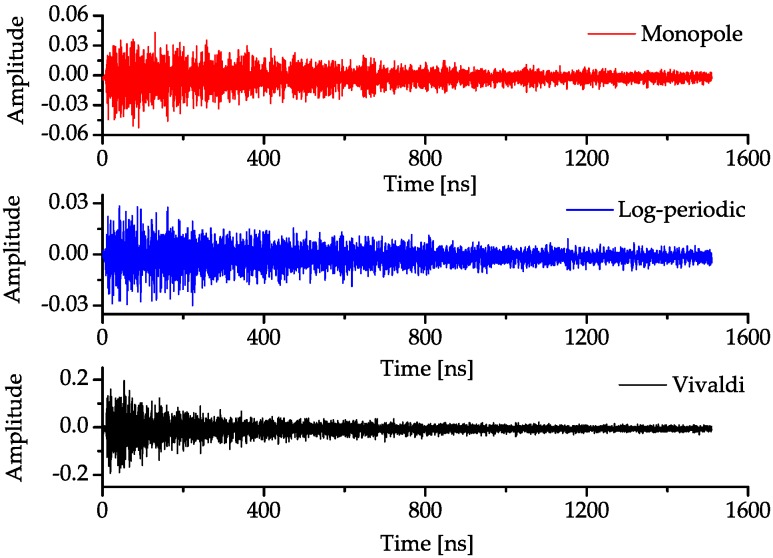
Original received signal after channel propagation with noise.

All the transmitted pulses in every measurement are of the same amplitude and time duration, and the original received signals during one measurement that contains noise are shown in Figure 13. In addition, the noise will then be filtered out using the LMS filter. A typical deconvolution algorithm called the CLEAN algorithm is used to calculate the transmission responses [22]. The template signal is the input Gaussian signal with bandwidth from 2 GHz to 3 GHz at the *Tx* antenna, and the received signal is the output signal at the *Rx* antenna. In this way, the measured response is the convolution of the antenna transfer functions and spatial channel responses. The algorithm stops when correlations within a threshold from the strongest path cannot be found. For these measurements, the onset of “phantom paths” as characterized by a sharp increase in both the total number of detected multipath components and delay spreads, which occurred for a threshold of 30 dB. A threshold of 28 dB produced a correlation of 91–96% for both monopole and log-periodic antennas and energy capture ratio of 89–95%. Moreover, a threshold of 26 dB produced a correlation of 90–95% and the energy capture ratio is 86–92% for the Vivaldi antenna.

In Figure 13, measured results show that the Vivaldi antenna has a maximum channel gain while the log-periodic antenna has a minimum gain on average. The main lobes of the log-periodic and Vivaldi antenna are along the *y*-axis while the main lobe of the monopole is along the *z*-axis. Therefore, the signals transmitted by the log-periodic and Vivaldi antenna will undergo more reflections before reaching the *Rx* antenna under most of the measurement conditions. Since the pulse that was transferred by the log-periodic antenna has a larger time spread, the signal energy will dispersed in a larger time duration, which means that the amplitude of the signal will become smaller. After long distance propagation, the signal with a smaller amplitude will be received with larger attenuation, and a part of the signals will be at the same level with the noise. This is the reason why the average channel gain of the system composed by log-periodic antennas is smaller than the system composed by the monopoles, even if the gain of the log-periodic antenna is much larger than the monopole. The energy of the radiated signal of monopole is more centralized than the log-periodic antenna, and the larger gain of the log-periodic antenna cannot compensate its dispersed signal energy in this case. Similarly, the dispersion of the Vivaldi antenna is larger than that of the monopole, but its larger gain can compensate for the dispersion of the radiated signal energy; thus, the average channel gain of the Vivaldi antenna system is the largest.

The stability of phase center in a UWB antenna is related to the radiation mechanism and feed structure employed to excite the antenna. The phase center of the Vivaldi antenna has the worst stability here due to its unsymmetrical feed structure, and it does not affect the intensity of the radiated signal. According to the above analysis, in the given measurement environment, the weakened signal intensity caused by the antenna dispersion is the main reason or the received small amplitude signals.

Thus, the stability of the phase center does not have a direct interconnection with the performance of the TR UWB communication systems.

**Figure 14:**
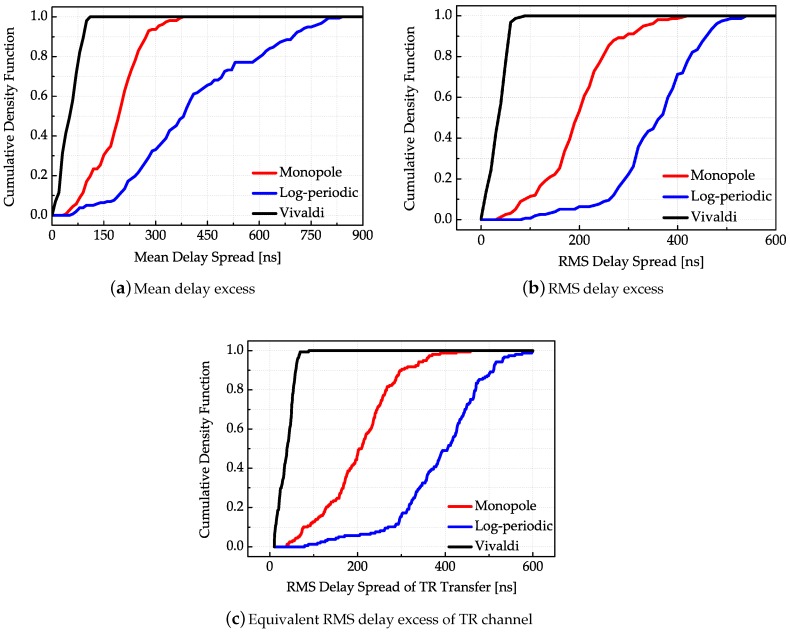
(**a**) Cumulative density functions of mean channel delay spreads; (**b**) cumulative density functions of the channel root-mean-square (RMS) delay spreads; (**c**) cumulative density functions of the equivalent TR channel RMS delay spreads corresponding to different antenna systems.

The power delay profile (PDP) is characterized by the first central moment (mean excess delay) and the square root of the second moment (root mean square (RMS) delay spread). The RMS delay provides a figure of merit for estimating data rates of the TR UWB sensors system [10] due to the fact that the equivalent TR channel response in time domain is just the self-convolution of the unidirectional transmission response. This means that, once the unidirectional response is obtained, the equivalent TR channel response can be correspondingly calculated. The cumulative density functions of the mean delay spread and RMS delay spread of the unidirectional and TR transmission are shown in Figure 14.

The results in Figure 14 show that the Vivaldi antenna has both the minimum mean delay spread and RMS delay spread. This is intuitive because of its largest channel gain. For the setting of the given threshold value, multiple paths of the Vivaldi antenna with extremely lower amplitudes are regarded as noise thus being filtered out. Similarly, the channel gain of the log-periodic antenna pair is relatively the smallest; thus, more multipath components are detected above a given threshold with larger path delay. Because of the temporal focusing effect of TR transmission, most of the energy is focused on the path with maximal transmission gain, the cumulative density functions of the mean excess delay corresponding to equivalent TR transmission are highly concentrated, but the RMS behaves differently by observing Figure 14c. Because of its overall highest transmission gain, the Vivaldi antenna has the smallest RMS delay spread in the TR transmission while the log-periodic antenna correspondingly has the largest RMS delay spread. The reason is the same as in the unidirectional transmission.

When the signal is detected at the receiver in pulse-based TR UWB sensor systems, only a threshold decision is needed to detect the information symbol and its value depends on the maximum transmission gain. All other taps of the equivalent TR transmission link in time domain can be regarded as interference. The larger the threshold, the better anti-interference performance the TR system possesses. In order to precisely describe the energy focusing performance that is closely related to the detection error rate and anti-interference performance of the TR system, we define here a metric that is signal-to-mean-interference ratio (SMIR). The SMIR is the ratio between the maximum equivalent TR transmission gain and the mean interference of other taps. Thus, according to (10)–(12), it can be denoted as $\mathrm{SMIR}=\left(2P-2\right)\cdot \int S_{gain}\left(f\right)/S_{int}\left(f\right)e^{j2\pi f}df$ in frequency domain. The SMIR performance can be calculated by the measured antenna transfer function and channel responses in frequency domain.

**Figure 15:**
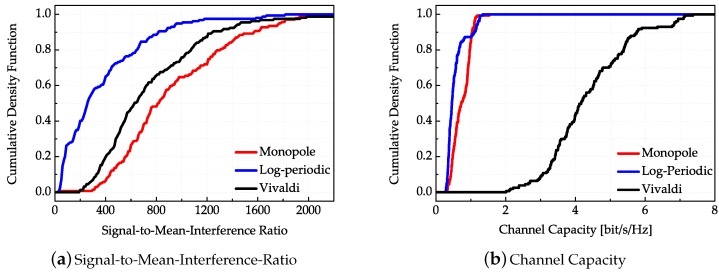
(**a**) Cumulative density functions of Signal-to-Nean-Interference-Ratio (SMIR) corresponding to the TR equivalent channels; (**b**) cumulative density functions of channel capacities corresponding to the TR system.

The cumulative density functions of the SMIR are exemplified in Figure 15a. The results show that the monopole antenna has the best SMIR performance while the log-periodic antenna has the worst SMIR performance. In other words, the monopole antenna has the best energy focusing ability and anti-interference performance while the log-periodic antenna possesses the worst energy focusing ability. Although the channel gains of the Vivaldi antenna are all relatively the largest, while the gains of the log-periodic antenna are the smallest, they have smaller ratios between the main path power and the average interference path power than the monopole antenna. The SMIR performances are in accordance with the antenna dispersion properties, which means that the smaller antenna dispersion can obtain better energy focusing property and anti-interference property. The cumulative density functions of evaluated channel capacities corresponding to each TR transmission are demonstrated in Figure 15b. Using the classical Shannon capacity formula, the measured time domain channel response is firstly turned into the frequency domain, and then the frequency band is divided into 1024 sub-bands, where each sub-channel can be considered as frequency-flat, and then integrate the channel capacity for the sub-bands over the whole bandwidth [23]. Through the total derivation procedure of the TR UWB system, the channel capacity $C_{\mathrm{TR}}$ of the TR system is given by

(15) $$C_{\mathrm{TR}}=\max_{\int_{-B/2}^{B/2}S_{X}\left(f\right)df=P_{t}}\int_{-B/2}^{B/2}log\left(1+\frac{S_{X}\left(f\right){∥H_{\mathrm{TR}}\left(f\right)∥}^{2}}{N_{0}}\right)df$$

In the above equation, *B* is the system bandwidth, and $P_{t}$ is the total transmitting signal power. Moreover, $H_{\mathrm{TR}}\left(f\right)$ is the TR transmission function in frequency domain and $S_{X}\left(f\right)/N_{0}$ is the SNR of each small sub-channel. The SNR is 5 dB in this computation. Unsurprisingly, the Vivaldi antenna pair has the maximum channel capacities while the log-periodic antenna pair has the minimum channel capacities that are consistent with the transmission channel gains.

## 6. Conclusions

Practical channel measurements about the TR UWB transmission link in a dense multipath environment are performed in order to investigate the effects of antennas on the performance of the sensors system. A detailed description of the deployed antennas, measurement setup, employed signal waveforms and data post-processing have been provided. Specific analysis including delay properties, SMIR parameters of the equivalent TR channel responses and channel capacities corresponding to different antennas have been presented. The antenna dispersion property will do harm to the SNR at the *Rx* antenna and decrease the channel capacity. Moreover, the antenna dispersion effect to some extent cannot be compensated by the larger antenna gain. Smaller time dispersion of the signal caused by antenna dispersion will have better energy focusing property in TR UWB communications. The results enable significant guidance for the trade-off selection between antenna dispersion and gain in subsequent TR UWB WSNs’ system deployment with low-complexity and low-cost receivers.

## Figures and Tables

**Table 1:** The corresponding maximal gain (dBi) and 3 dB beamwidth (${}^{\circ }$) of three antennas on different planes with frequencies 2 GHz or 3 GHz.

Gain (dBi)—Beamwidth	Monopole (2 GHz/3 GHz)	Log-periodic (2 GHz/3 GHz)	Vivaldi (2 GHz/3 GHz)
*xoy* plane	0.9–$90.2^{\circ }$/0.2–$93.7^{\circ }$	8.9–$62.2^{\circ }$/7.2–$76.9^{\circ }$	6.2–$83.8^{\circ }$/9.1–$47.5^{\circ }$
*yoz* plane	1.9–$360^{\circ }$/2.3–$360^{\circ }$	8.9–$87^{\circ }$/7.5–$107.4^{\circ }$	6.2–$73^{\circ }$/9.1–$70.2^{\circ }$

## Abbreviations

The following abbreviations are used in this manuscript:

UWB	Ultra-wideband
TR	Time reversal
WSN	Wireless sensors network
SNR	Signal-to-noise ratio
Tx	Transmitter
Rx	Receiver
VNA	Vector network analyzer
AWG	Arbitrary waveform generator
DSA	Digital serial analyzer
PA	Power amplifier
LMS	Least-mean-square
RMS	Root mean square
PDP	Power delay profile
SMIR	Signal-to-mean-interference ratio
